# Supplementary material for: HIVprotI: an integrated web based platform for prediction and design of HIV proteins inhibitors
Source: J Cheminform. 2018 Mar 9;10:12. doi: 10.1186/s13321-018-0266-y (PMC5845081; doi:10.1186/s13321-018-0266-y)
Supplement: Supplementary file 2 — Additional file 2. Source code of HIVProtI web server. [file 13321_2018_266_MOESM2_ESM.zip › HIVprotI_Source-code/hivprotI/weka-3-6-6/doc/weka/core/xml/XMLBasicSerialization.html]

XMLBasicSerialization


---


|  |  |  |
| --- | --- | --- |
| **Overview** \| **Package** \| **Class** \| **Tree** \| **Deprecated** \| **Index** \| **Help** \| **Weka's home** | |  |
| **PREV CLASS**   **NEXT CLASS** | **FRAMES**    **NO FRAMES**     **All Classes** |  |
| SUMMARY: NESTED \| FIELD \| CONSTR \| METHOD | DETAIL: FIELD \| CONSTR \| METHOD |  |


---


## weka.core.xml Class XMLBasicSerialization

```
java.lang.Object
  weka.core.xml.XMLSerialization
      weka.core.xml.XMLBasicSerialization
```

**All Implemented Interfaces:**: RevisionHandler

**Direct Known Subclasses:**: XMLBeans, XMLClassifier, XMLExperiment

---

``` public class XMLBasicSerialization extends XMLSerialization ```

This serializer contains some read/write methods for common classes that
are not beans-conform. Currently supported are:

- java.util.HashMap
- java.util.HashSet
- java.util.Hashtable
- java.util.LinkedList
- java.util.Properties
- java.util.Stack
- java.util.TreeMap
- java.util.TreeSet
- java.util.Vector
- javax.swing.DefaultListModel

Weka classes:

- weka.core.Matrix
- weka.core.matrix.Matrix

**Version:**
:   $Revision: 1.6 $

**Author:**
:   FracPete (fracpete at waikato dot ac dot nz)

---

| **Field Summary** | |
| --- | --- |
| `static java.lang.String` | `VAL_CELLS`             the matrix cells |
| `static java.lang.String` | `VAL_KEY`             the value for a mapping-key, e.g., Maps |
| `static java.lang.String` | `VAL_MAPPING`             the value for mapping, e.g., Maps |
| `static java.lang.String` | `VAL_VALUE`             the value for mapping-value, e.g., Maps |

**Fields inherited from class weka.core.xml.XMLSerialization**

`ATT_ARRAY, ATT_ARRAY_DEFAULT, ATT_CLASS, ATT_NAME, ATT_NULL, ATT_NULL_DEFAULT, ATT_PRIMITIVE, ATT_PRIMITIVE_DEFAULT, ATT_VERSION, DOCTYPE, ROOT_NODE, TAG_OBJECT, VAL_NO, VAL_ROOT, VAL_YES`


**Constructor Summary**

`XMLBasicSerialization()`
  
          initializes the serialization


| **Method Summary** | |
| --- | --- |
| `void` | `clear()`             generates internally a new XML document and clears also the IgnoreList and the mappings for the Read/Write-Methods |
| `java.lang.String` | `getRevision()`             Returns the revision string. |
| `java.lang.Object` | `readCollection(org.w3c.dom.Element node)`             builds the Collection from the given DOM node. |
| `java.lang.Object` | `readCostMatrixOld(org.w3c.dom.Element node)`             builds the Matrix (old) from the given DOM node. |
| `java.lang.Object` | `readDefaultListModel(org.w3c.dom.Element node)`             builds the DefaultListModel from the given DOM node. |
| `java.lang.Object` | `readMap(org.w3c.dom.Element node)`             builds the Map from the given DOM node. |
| `java.lang.Object` | `readMatrix(org.w3c.dom.Element node)`             builds the Matrix from the given DOM node. |
| `java.lang.Object` | `readMatrixOld(org.w3c.dom.Element node)`             builds the Matrix (old) from the given DOM node. |
| `org.w3c.dom.Element` | `writeCollection(org.w3c.dom.Element parent, java.lang.Object o, java.lang.String name)`             adds the given Collection to a DOM structure. |
| `org.w3c.dom.Element` | `writeCostMatrixOld(org.w3c.dom.Element parent, java.lang.Object o, java.lang.String name)`             adds the given CostMatrix (old) to a DOM structure. |
| `org.w3c.dom.Element` | `writeDefaultListModel(org.w3c.dom.Element parent, java.lang.Object o, java.lang.String name)`             adds the given DefaultListModel to a DOM structure. |
| `org.w3c.dom.Element` | `writeMap(org.w3c.dom.Element parent, java.lang.Object o, java.lang.String name)`             adds the given Map to a DOM structure. |
| `org.w3c.dom.Element` | `writeMatrix(org.w3c.dom.Element parent, java.lang.Object o, java.lang.String name)`             adds the given Matrix to a DOM structure. |
| `org.w3c.dom.Element` | `writeMatrixOld(org.w3c.dom.Element parent, java.lang.Object o, java.lang.String name)`             adds the given Matrix (old) to a DOM structure. |

**Methods inherited from class weka.core.xml.XMLSerialization**

`fromXML, getVersion, main, read, read, read, read, readBooleanFromXML, readByteFromXML, readCharFromXML, readDoubleFromXML, readFloatFromXML, readFromXML, readFromXML, readIntFromXML, readLongFromXML, readShortFromXML, toXML, write, write, write, write, writeToXML`

**Methods inherited from class java.lang.Object**

`equals, getClass, hashCode, notify, notifyAll, toString, wait, wait, wait`

**Field Detail**

### VAL\_MAPPING

```
public static final java.lang.String VAL_MAPPING
```

:   the value for mapping, e.g., Maps

    **See Also:**: Constant Field Values

---


### VAL\_KEY

```
public static final java.lang.String VAL_KEY
```

:   the value for a mapping-key, e.g., Maps

    **See Also:**: Constant Field Values

---


### VAL\_VALUE

```
public static final java.lang.String VAL_VALUE
```

:   the value for mapping-value, e.g., Maps

    **See Also:**: Constant Field Values

---


### VAL\_CELLS

```
public static final java.lang.String VAL_CELLS
```

:   the matrix cells

    **See Also:**: Constant Field Values


**Constructor Detail**

### XMLBasicSerialization

```
public XMLBasicSerialization()
                      throws java.lang.Exception
```

:   initializes the serialization

    **Throws:**: `java.lang.Exception` - if initialization fails


**Method Detail**

### clear

```
public void clear()
           throws java.lang.Exception
```

:   generates internally a new XML document and clears also the IgnoreList
    and the mappings for the Read/Write-Methods

    :   **Overrides:**: `clear` in class `XMLSerialization`
    :   **Throws:**: `java.lang.Exception` - if initializing fails

---


### writeDefaultListModel

```
public org.w3c.dom.Element writeDefaultListModel(org.w3c.dom.Element parent,
                                                 java.lang.Object o,
                                                 java.lang.String name)
                                          throws java.lang.Exception
```

:   adds the given DefaultListModel to a DOM structure.

    :   **Parameters:**: `parent` - the parent of this object, e.g. the class this object is a member of: `o` - the Object to describe in XML: `name` - the name of the object **Returns:**: the node that was created **Throws:**: `java.lang.Exception` - if the DOM creation fails **See Also:**: `DefaultListModel`

---


### readDefaultListModel

```
public java.lang.Object readDefaultListModel(org.w3c.dom.Element node)
                                      throws java.lang.Exception
```

:   builds the DefaultListModel from the given DOM node.

    :   **Parameters:**: `node` - the associated XML node **Returns:**: the instance created from the XML description **Throws:**: `java.lang.Exception` - if instantiation fails **See Also:**: `DefaultListModel`

---


### writeCollection

```
public org.w3c.dom.Element writeCollection(org.w3c.dom.Element parent,
                                           java.lang.Object o,
                                           java.lang.String name)
                                    throws java.lang.Exception
```

:   adds the given Collection to a DOM structure.

    :   **Parameters:**: `parent` - the parent of this object, e.g. the class this object is a member of: `o` - the Object to describe in XML: `name` - the name of the object **Returns:**: the node that was created **Throws:**: `java.lang.Exception` - if the DOM creation fails **See Also:**: `Collection`

---


### readCollection

```
public java.lang.Object readCollection(org.w3c.dom.Element node)
                                throws java.lang.Exception
```

:   builds the Collection from the given DOM node.

    :   **Parameters:**: `node` - the associated XML node **Returns:**: the instance created from the XML description **Throws:**: `java.lang.Exception` - if instantiation fails **See Also:**: `Collection`

---


### writeMap

```
public org.w3c.dom.Element writeMap(org.w3c.dom.Element parent,
                                    java.lang.Object o,
                                    java.lang.String name)
                             throws java.lang.Exception
```

:   adds the given Map to a DOM structure.

    :   **Parameters:**: `parent` - the parent of this object, e.g. the class this object is a member of: `o` - the Object to describe in XML: `name` - the name of the object **Returns:**: the node that was created **Throws:**: `java.lang.Exception` - if the DOM creation fails **See Also:**: `Map`

---


### readMap

```
public java.lang.Object readMap(org.w3c.dom.Element node)
                         throws java.lang.Exception
```

:   builds the Map from the given DOM node.

    :   **Parameters:**: `node` - the associated XML node **Returns:**: the instance created from the XML description **Throws:**: `java.lang.Exception` - if instantiation fails **See Also:**: `Map`

---


### writeMatrix

```
public org.w3c.dom.Element writeMatrix(org.w3c.dom.Element parent,
                                       java.lang.Object o,
                                       java.lang.String name)
                                throws java.lang.Exception
```

:   adds the given Matrix to a DOM structure.

    :   **Parameters:**: `parent` - the parent of this object, e.g. the class this object is a member of: `o` - the Object to describe in XML: `name` - the name of the object **Returns:**: the node that was created **Throws:**: `java.lang.Exception` - if the DOM creation fails **See Also:**: `Matrix`

---


### readMatrix

```
public java.lang.Object readMatrix(org.w3c.dom.Element node)
                            throws java.lang.Exception
```

:   builds the Matrix from the given DOM node.

    :   **Parameters:**: `node` - the associated XML node **Returns:**: the instance created from the XML description **Throws:**: `java.lang.Exception` - if instantiation fails **See Also:**: `Matrix`

---


### writeMatrixOld

```
public org.w3c.dom.Element writeMatrixOld(org.w3c.dom.Element parent,
                                          java.lang.Object o,
                                          java.lang.String name)
                                   throws java.lang.Exception
```

:   adds the given Matrix (old) to a DOM structure.

    :   **Parameters:**: `parent` - the parent of this object, e.g. the class this object is a member of: `o` - the Object to describe in XML: `name` - the name of the object **Returns:**: the node that was created **Throws:**: `java.lang.Exception` - if the DOM creation fails **See Also:**: `Matrix`

---


### readMatrixOld

```
public java.lang.Object readMatrixOld(org.w3c.dom.Element node)
                               throws java.lang.Exception
```

:   builds the Matrix (old) from the given DOM node.

    :   **Parameters:**: `node` - the associated XML node **Returns:**: the instance created from the XML description **Throws:**: `java.lang.Exception` - if instantiation fails **See Also:**: `Matrix`

---


### writeCostMatrixOld

```
public org.w3c.dom.Element writeCostMatrixOld(org.w3c.dom.Element parent,
                                              java.lang.Object o,
                                              java.lang.String name)
                                       throws java.lang.Exception
```

:   adds the given CostMatrix (old) to a DOM structure.

    :   **Parameters:**: `parent` - the parent of this object, e.g. the class this object is a member of: `o` - the Object to describe in XML: `name` - the name of the object **Returns:**: the node that was created **Throws:**: `java.lang.Exception` - if the DOM creation fails **See Also:**: `CostMatrix`

---


### readCostMatrixOld

```
public java.lang.Object readCostMatrixOld(org.w3c.dom.Element node)
                                   throws java.lang.Exception
```

:   builds the Matrix (old) from the given DOM node.

    :   **Parameters:**: `node` - the associated XML node **Returns:**: the instance created from the XML description **Throws:**: `java.lang.Exception` - if instantiation fails **See Also:**: `CostMatrix`

---


### getRevision

```
public java.lang.String getRevision()
```

:   Returns the revision string.

    :   **Specified by:**: `getRevision` in interface `RevisionHandler` **Overrides:**: `getRevision` in class `XMLSerialization`
    :   **Returns:**: the revision


---


|  |  |  |
| --- | --- | --- |
| **Overview** \| **Package** \| **Class** \| **Tree** \| **Deprecated** \| **Index** \| **Help** \| **Weka's home** | |  |
| **PREV CLASS**   **NEXT CLASS** | **FRAMES**    **NO FRAMES**     **All Classes** |  |
| SUMMARY: NESTED \| FIELD \| CONSTR \| METHOD | DETAIL: FIELD \| CONSTR \| METHOD |  |


---
